# Mapping threatened Thai bovids provides opportunities for improved conservation outcomes in Asia

**DOI:** 10.1098/rsos.240574

**Published:** 2024-09-25

**Authors:** Wantida Horpiencharoen, Renata L. Muylaert, Jonathan C. Marshall, Reju Sam John, Antony J. Lynam, Alex Riggio, Alexander Godfrey, Dusit Ngoprasert, George A. Gale, Eric Ash, Francesco Bisi, Giacomo Cremonesi, Gopalasamy Reuben Clements, Marnoch Yindee, Nay Myo Shwe, Chanratana Pin, Thomas N. E. Gray, Saw Soe Aung, Seree Nakbun, Stephanie G. Manka, Robert Steinmetz, Rungnapa Phoonjampa, Naret Seuaturien, Worrapan Phumanee, David T. S. Hayman

**Affiliations:** ^1^ Molecular Epidemiology and Public Health Laboratory, Hopkirk Research Institute, Massey University, Palmerston North 4472, New Zealand; ^2^ Centre for Global Conservation, Wildlife Conservation Society, New York, NY, USA; ^3^ Mahidol University, Salaya, Phutthamonthon, Nakhon Pathom, Thailand; ^4^ Panthera, 8 West 40th Street 18th Floor, New York, NY 10018, USA; ^5^ Conservation Ecology Program, School of Bioresources and Technlogy, King Mongkut’s University of Technology Thonburi, Bangkok, Thailand; ^6^ Wildlife Conservation Research Unit, University of Oxford, Oxford, UK; ^7^ Environmental Analysis and Management Unit - Guido Tosi Research Group - Department of Theoretical and Applied Sciences, University of Insubria, Varese, Italy; ^8^ Istituto Oikos E.T.S., Milan, Italy; ^9^ Zoological Society of London, Regents Park, London, UK; ^10^ Akkhraratchakumari Veterinary College, Walailak University, Nakhon Si Thammarat 80160, Thailand; ^11^ Friends of Wildlife, Yan-Aung Street (1), Building 296, Room 15 Quarter No. (2), Yankin Township, Yangon, Myanmar; ^12^ Ministry of Environment, 48 Samdach Preah Sihanouk Blvd., Phnom Penh 12301, Cambodia; ^13^ WWF Tigers Alive Initiative, Phnom Penh, Cambodia; ^14^ Fauna & Flora International, Myanmar Programme, Bahan Township, Yangon, Myanmar; ^15^ Khaonampu Nature and Wildlife Education Center, Department of National Park, Wildlife and Plant Conservation, Kanchanaburi, Thailand; ^16^ Fancy Scientist LLC, Glen Ellyn, IL, USA; ^17^ WWF-Thailand, 9 Pisit Building, Pradiphat Road Soi 10, Phayathai, Bangkok 10400, Thailand

**Keywords:** species distribution, herbivores, large mammals, wildlife conservation, habitat suitability, protected areas

## Abstract

Wild bovids provide important ecosystem functions as seed dispersers and vegetation modifiers. Five wild bovids remain in Thailand: gaur (*Bos gaurus*), banteng (*Bos javanicus*), wild water buffalo (*Bubalus arnee*), mainland serow (*Capricornis sumatraensis*) and Chinese goral (*Naemorhedus griseus*). Their populations and habitats have declined substantially and become fragmented by land-use change. We use ecological niche models to quantify how much potential suitable habitat for these species remains within protected areas in Asia and then specifically Thailand. We combined species occurrence data from several sources (e.g. mainly camera traps and direct observation) with environmental variables and species-specific and single, large accessible areas in ensemble models to generate suitability maps, using out-of-sample predictions to validate model performance against new independent data. Gaur, banteng and buffalo models showed reasonable model accuracy throughout the entire distribution (greater than or equal to 62%) and in Thailand (greater than or equal to 80%), whereas serow and goral models performed poorly for the entire distribution and in Thailand, though 5 km movement buffers markedly improved the performance for serow. Large suitable areas were identified in Thailand and India for gaur, Cambodia and Thailand for banteng and India for buffalo. Over 50% of suitable habitat is located outside protected areas, highlighting the need for habitat management and conflict mitigation outside protected areas.

## 1. Introduction

An important task of wildlife research and conservation is to define the distributional ecology of species and to understand how they relate to the environment, climate and other organisms [1]. Ecological niche models (ENM) are used to predict the geographic suitability of a species by using ecological niche dimensions combined with species’ presence data [2]. ENM can be approached using the ‘biotic–abiotic–mobility’ (BAM) framework, which considers the relationship between the species’ distribution, and geographical and climatic factors, and explains the influence of these factors on predicted habitat suitability [3]. Abiotic (A) factors generally determine the potential distribution (or fundamental niche) of a species, and the intersection of abiotic and biotic (B) factors form the realized niche, or the part of this potential distribution where species actually live [4]. Mobility (M) is the area accessible by species related to their distribution over periods of time (the ‘accessible area’ [5]). Selecting the extent of species’ accessible areas, including buffer zones, impacts model prediction results [5,6].

Wild Bovidae (Mammalia: Artiodactyla) play significant ecological roles in tropical forests and grasslands [7] as grazers and browsers, by modifying plant diversity and abundance within ecosystems [8,9]. Large wild bovids are also the prey of predators such as tigers (*Panthera tigris*) and leopards (*P. pardus*) [10]. Throughout Asia, wild bovid populations are threatened by poaching [11] and habitat loss [12], especially in South to Southeast Asia [13]. Natural habitats have been disturbed by free-grazing livestock, which can lead to interbreeding (e.g. between domestic and wild water buffalo) [14], increased competition for food and natural resources [15] and increased risk of disease transmission between wildlife and livestock [16,17]. Moreover, habitat destruction is likely to influence the species’ distribution and behaviour adaptation, which could lead to shared natural resources and conflict between humans and wild bovids.

**Figure 1.**
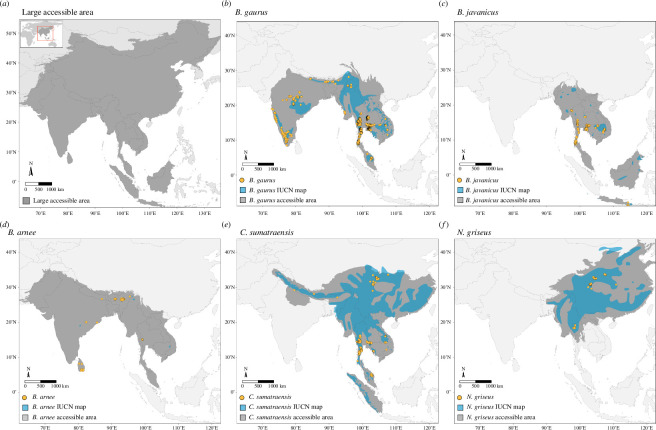
Species occurrence data before thinning (yellow circles), IUCN polygons (blue areas) and study areas (grey areas) used in model building for five wild bovid species. First, a common large ‘accessible area’ (*a*) was used for all species for model building, and then species-specific accessible areas (*b–f*) for individual species.

In South and Southeast Asia, there are 27 recognized bovid species [18], of which seven species are listed as vulnerable, five as endangered and three as critically endangered with extinction. Thailand has five bovid species (gaur*: Bos gaurus*; banteng: *Bos javanicus*; wild water buffalo: *Bubalus arnee*; mainland serow: *Capricornis sumatraensis* and Chinese goral: *Naemorhedus griseus*) remaining in their natural habitat. These species are distributed in other countries from South to Southeast Asia (figure 1) and also have different suitable habitats. For example, gaur can be found in evergreen forests or grassland and range from India, Nepal, across Southeast Asia (SEA) to Peninsula Malaysia [19]. Mainland serow also has a wide distribution from Nepal to Sumatra in Indonesia through hill forests to shrubland habitats [20]. Nevertheless, the prediction of the remaining habitat quality and suitability in Thailand and other countries have been conducted only in some protected areas [21,22], but not at the regional or national level.

Species distribution modelling provides an overview of potential habitats for threatened species and aids in conservation planning [23]. For instance, previous studies have focused on identifying potentially high-quality habitat connectivity and fragmentation [24] as well as predicting global biodiversity trends [25]. In Thailand, there are several studies that have predicted habitat suitability for some of these five wild bovids in local areas [26], but habitat suitability studies for larger extents across their distribution are lacking.

Here, we built ENMs for the five Thai wild bovid species: gaur, banteng, wild water buffalo, mainland serow and Chinese goral at two scales: first, at the regional scale throughout the entire distribution and, second, at the country scale in Thailand. We aim to (i) identify the potential distribution for these five species in South to Southeast Asia, and (ii) identify conservation areas in their geographical distribution, with a particular focus on Thailand.

## 2. Material and methods

Our workflow consisted of two main processes of data preparation and model building (summarized in electronic supplementary material, figure S1) that generated habitat suitability maps for all species and accessible areas used. Data preparation consisted of gathering the species occurrence data and environmental data and selecting the accessible areas (see the following text). Then, the model building consisted of pre-processing, processing and post-processing steps.

### 2.1. Study area

The study area consists of 13 Asian countries: Bhutan, Bangladesh, Cambodia, China, India, Indonesia, Laos, Malaysia, Myanmar, Nepal, Sri Lanka, Thailand and Vietnam (figure 1), which cover the distributions of gaur, banteng, wild water buffalo, mainland serow and Chinese goral based on the literature (electronic supplementary material, table S1).

### 2.2. Species occurrence data

Raw species occurrence data came from several different sources. The data were collected using various field study methodologies, including observation of animal signs (e.g. footprint and dung; 23 396 points) during forest patrols, direct observation during wildlife surveys (444 points), camera trapping (5483 points), and radio-collar signals (4341 points), comprising data collected from 23 organizations (electronic supplementary material, table S2). We used species occurrence data from GPS records collected between January 2000 and June 2021 from researchers, government, NGOs (World Wildlife Fund, Wildlife Conservation Society, Freeland [27], Panthera, Fauna & Flora International, Friends of Wildlife and RIMBA) and open data sources, including GBIF (https://www.gbif.org/) and eMammal (https://emammal.si.edu/). The data coverage by country can be found in table 1, and for details of the data collection sites, see the electronic supplementary material, S10.

**Table 1.** The number of raw and after spatial thinning occurrence points is shown by species and country.

Country	*Bos gaurus*	*Bos javanicus*	*Bubalus arnee*	*Capricornis sumatraensis*	*Naemorhedus griseus*	Total
raw data						
Bangladesh	X	X^c^	X	?^b^	X	0
Bhutan	1^a^	X	?	?	X	1
Cambodia	44	355	?	38	X	437
China	?	?	X	109	301	410
India	286	X	78	2	?	366
Indonesia	X	6	X	?	X	6
Laos	2	1	X	11	X	14
Malaysia	1067	?	X	603	X	1671
Myanmar	114	5	?	99	?	218
Nepal	4	X	1	2	X	7
Sri Lanka	X	X	21	X	X	21
Thailand	24 258	5383	50	805	16	30 512
Vietnam	1	?	?	?	?	1
**grand total**	**25 777**	**5751**	**150**	**1669**	**317**	**33 664**
thinned data						
Bangladesh	X	X	X	?	X	
Bhutan	1	X	?	?	X	1
Cambodia	14	48	?	28	X	90
China	?	?	X	64	130	194
India	244	X	64	2	?	310
Indonesia	X	6	X	?	X	6
Laos	2	1	X	8	X	11
Malaysia	26	1	X	49	X	76
Myanmar	53	2	?	51	?	106
Nepal	4	X	1	1	X	6
Sri Lanka	X	X	20	X	X	20
Thailand	2387	303	7	185	5	2887
Vietnam	1	?	?	?	?	1
**grand total**	**2732**	**361**	**92**	**388**	**135**	**3708**

^a^ number- species presence with occurrence data

^b^ ?- species presence without occurrence data

^c^ X- no species presence

We used only the research grade observations for GBIF data, which included a photo for species identification. We filtered all the occurrences and excluded occurrence records outside the species-specific accessible area, and duplicated records from the same species and museum collections.

### 2.3. Environmental variables

Hypothesized environmental variables were selected based on species’ habitat and distribution-related literature (electronic supplementary material, table S1). We used 28 variables (electronic supplementary material, table S3) for model construction, including 19 bioclimatic variables [28] (the average for 1970–2000) from WorldClim v. 2 [29], elevation (Shuttle Radar Topography Mission, SRTM) from WorldClim [29], slope [30], five land cover fractions (grass, tree, urban, water and crop) [31], human population density [32] and greenness through the normalized difference vegetation index (NDVI). All layers were processed using the geographic coordinates system (Datum WGS84) at approximately 1 km^2^ spatial resolution. We transformed the skewed human population density using logarithm base 10. We rescaled the NDVI layer by multiplying all values with a scale factor (0.0001), based on the Moderate Resolution Imaging Spectroradiometer (MODIS) user’s guidelines [33].

### 2.4. Accessible areas

The accessible area refers to the parts of the world accessible to species via dispersal over time [5]. The extent of the accessible area and the inclusion of a buffer zone have an important effect on ENM performance [5,6]. We used two accessible area sizes to delimitate our modelling extent (figure 1). The first larger accessible area (hereon LA) includes most of the Asian continent and its ecoregions, and all species distributions are included as a common extent. The second accessible area was more restricted and cropped based on individual species-specific distributions (hereon SSA) from literature reviews (electronic supplementary material, table S1), IUCN polygons or ‘ranges’ [34] and the terrestrial ecoregions where they occur.

To create the extent, we downloaded the current IUCN range maps for each species, then intersected those over ecoregions [35], and then combined the results with selected ecoregions based on biogeographic knowledge of the species distributions and habitat preference from the literature reviews. For example, the gaur habitat typically contains moist evergreen, semi-evergreen and dry evergreen forests [36,37], so we included these regions in our accessible areas. Further details on ecoregions included in accessible areas are in supplementary materials (electronic supplementary material, table S4). To reduce overprediction and make our predictions closer to realized niche estimates, we used an occurrences-based threshold (OBR) method with ensemble models from [38] for creating the spatially restricted ENM (hereon MSDM). OBR is an *a posteriori* method that restricts the suitable areas of our final ensemble models based on presence and the largest nearest neighbour distance among pairs of occurrences. Overall, we built four combinations between two accessible areas with and without MSDM methods for each species, comprising (i) No MSDM-SSA, (ii) No MSDM-LA, (iii) MSDM-SSA, and (iv) MSDM-LA.

### 2.5. Model building

We processed the species occurrence files and environmental datasets in R 4.0.1 [39]. We developed reproducible ecological niche models with optimized processing times using the ENMTML package [40], following three main steps: (i) pre-processing, (ii) processing, and (iii) post-processing.

In pre-processing, we performed occurrence thinning using 2 times the cell size (1 km^2^) [41] to reduce clustering of species records and sampling bias. We used principal component (PC) analysis (PCA) to reduce the collinearity of the predictors. We assigned species’ accessible areas to determine the species’ distributions using a mask function. We used random sampling to create pseudo-absence background points in a 1 : 1 ratio with presence points [42]. The occurrence and pseudo-absence data were divided into two sets for fitting the model (75%) and evaluating the fitted models (25%), using the bootstrapping partition method with 10 replications for each algorithm.

In the processing step, eight algorithms were used to build the ENMs, namely: BIOCLIM [28], generalized linear models [43], generalized additive models [44], random forest [45], support vector machine [46], maximum entropy default [47], maximum likelihood [48] and Bayesian Gaussian process [49]. All models used the default settings from the ENMTML package, which included the functions from different packages (e.g. dismo, maxnet) based on the algorithms that are used to fit the models. The data type used for each algorithm is in electronic supplementary material, table S5.

In the post-processing step, we created ensemble models using the weighted average (WMEAN) method based on the true skill statistic (TSS) values for building final habitat suitability and binary maps. The benefits of ensemble models are (i) robust decision-making [50]; (ii) reducing uncertainty [51]; and (iii) a combination of several models into one model prediction [52]. We used TSS to calculate threshold values to convert habitat suitability maps into binary suitability maps (0 = unsuitable and 1 = suitable). We used TSS and ‘area under the curve’ (AUC) values for evaluating model performance. The TSS threshold is calculated using the maximum summed specificity and sensitivity and is not based on prevalence, where an equal TSS score for given models means similar performance [53]. Therefore, we selected the final models from the best TSS of weighted average ensemble models. We assessed the model’s accuracy by plotting a new dataset of species occurrences obtained from camera traps and human observations (https://www.gbif.org/) on the binary maps. Because, for example, gaur have been recorded to walk up to 6.3 km per day (mean 1.6 km [54]), we created a 5 km buffer zone measured from the edges of the suitable pixels to include occurrences within the travel distance of wild bovids’ movement [55,56]. The percentage of points inside and outside the suitable areas and the buffer zone was calculated for each species. We first present all the results, then only select models with high prediction accuracy (greater than 80% [57]) for further analyses. The total suitable areas of the best TSS binary map models were calculated using the zonal function in the raster R package [58]. Then, we summed the pixels of the best TSS binary maps to generate the map of species number.

### 2.6. Protected area analyses

The source for our protected areas map was the World Database of Protected Area (WDPA) [59]. We classified protected areas based on IUCN protected areas from WPDA into eight categories, including categories 1 to 6 as IUCN management categories I–VI; category 7 as ‘not applicable’, which includes ‘not reported’, ‘not applicable’ and ‘not assigned’ protected areas; and category 8 as non-protected areas, which are the remaining areas that have not been classified as IUCN categories 1–7 [59]. Then, we used the zonal function in the raster package to calculate overlapping areas between the suitable areas and protected areas for each species.

We calculated the percentage of suitable areas in WDPA polygons using the exact_extract function in the exactextractr package [60] for extracting the suitable areas (values = 1) from the best TSS binary map rasters in each WDPA polygon. Then, we classified each PA into five different suitability categories based on the percentage of suitable habitat in the PA: low suitability (0%–20%); low to medium suitability (20%–40%); medium suitability (40%–60%); high suitability (60%–80%) and very high suitability (greater than or equal to 80%), and selected only the PAs that have suitable areas larger than species home range in the result. We used the published literature to identify the following home ranges: for gaur, 60 km^2^; for banteng, 45 km^2^ [61]; for wild water buffalo, 55 km^2^ [62]; 1 km^2^ for mainland serow [63]; and for Chinese, goral 6 km^2^ [64]. We have provided the code for creating the models in a GitHub repository and non-public data are available upon request.

## 3. Results

We compiled 33 664 occurrence records, then after filtering and spatial thinning, we used 3708 points for modelling: 2732 for gaur, 361 for banteng, 92 for wild water buffalo, 388 for mainland serow and 135 for Chinese goral. The majority of the thinned occurrences (77%) were collected in Thailand, India and other countries in mainland SEA; see table 1 for details on the data coverage by country and electronic supplementary material, table S10 for details on the study sites.

The PCA reduced the 28 environmental variables into 12 PCs that explained 95% of the environmental variance in the variables for the LA models for all species. The PCs for SSA models explained more than 96% of the total variance and the PC number varied by species, comprising 13 PCs (wild water buffalo), 11 PCs (gaur, mainland serow) and 10 PCs (banteng, Chinese goral). The bioclimatic variables were important variables in all species models. For LA models, the first two axes (PC1 and PC2) had high contributions from the annual mean temperature (bio01), the mean temperature of the coldest month (bio06), mean temperature of the driest quarter (bio09) and mean temperature of the warmest quarter (bio10). The first two axes of SSA models showed high positive contributions from the mean temperature of the coldest month (gaur), minimum temperature of the coldest month (banteng, mainland serow), annual mean temperature (wild water buffalo, mainland serow), and precipitation of the wettest quarter (Chinese goral). We found NDVI, elevation, slope and human population density have less effect on explaining the variability for the first two PCs for all species. The correlations between PCs and individual environmental variables, PC biplots and percentage of explained variance are summarized in electronic supplementary material, table S6 and figure S2.

### 3.1. Ecological niche models

Overall, all ensemble models showed high performance both for TSS and AUC with the best-performing models scoring over 0.8 for all species (table 2). Models with species-specific accessible areas were not always the best-performing models, but most ensemble models performed above 0.7 TSS. The habitat suitability prediction maps using the best model ensembles are in electronic supplementary material, figures S3 (SSA); S4 (LA); S5 (selected best SSA and LA models), and S6 (the binary maps which were used for calculating the suitable area). The performance of spatially restricted MSDM ensembles was higher in comparison with the No MSDM models, as the TSS was improved for banteng, Chinese goral and wild water buffalo. The lowest performing model for wild water buffalo was the No MSDM-SSA (TSS = 0.57). The best model for gaur was the No MSDM-LA model, banteng and Chinese goral the MSDM-LA model, wild water buffalo the MSDM-SSA model, and mainland serow the No MSDM-SSA model. We found that all species have small predicted suitable habitats. Moreover, all species’ models predicted less than 50% of the suitable areas inside PAs.

**Table 2.** TSS and AUC values of the weighted average ensemble model results, and the threshold values for binary maps for five species classified by accessible area type and MSDM method.

species	large accessible area						species-specific accessible area					
	no MSDM^a^			MSDM (OBR)^b^			no MSDM^a^			MSDM (OBR)^b^		
	TSS		AUC	TSS		AUC	TSS		AUC	TSS		AUC
	score	threshold	score	score	threshold	score	score	threshold	score	score	threshold	score
gaur *Bos gaurus*	**0.92**	0.49	0.99	0.92	0.44	0.99	**0.88**	0.39	0.98	0.88	0.41	0.98
banteng *Bos javanicus*	0.93	0.55	0.99	**0.94**	0.41	1	0.85	0.33	0.96	**0.83**	0.42	0.97
wild water buffalo *Bubalus arnee*	0.67	0.47	0.88	**0.72**	0.6	0.9	0.57	0.58	0.83	**0.85**	0.44	0.95
mainland serow *Capricornis sumatraensis*	**0.87**	0.55	0.97	0.76	0.47	0.94	**0.93**	0.57	0.98	0.93	0.52	0.98
Chinese goral *Naemorhedus griseus*	0.91	0.29	0.98	**0.91**	0.59	0.98	0.87	0.47	0.96	**0.9**	0.39	0.97

Best-performing models for each accessible area by TSS are shown in **boldface**.

^a^ spatially restricted ENM

^b^ occurrences-based threshold

**Figure 2.**
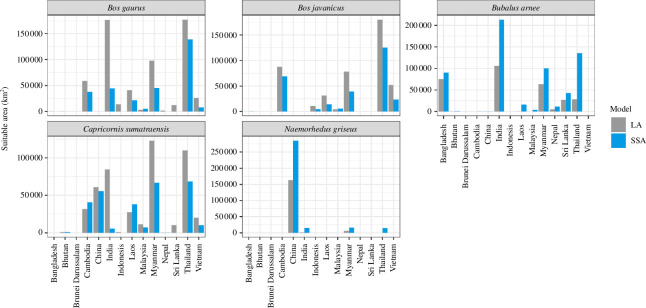
The predicted suitable areas in km^2^ for each species and country. Blue is the species-specific accessible area (SSA) and grey is the large accessible area models (LA) (see details in the electronic supplementary material, table S7).

The total predicted suitable areas in km^2^ for each species and country are shown in figure 2 and suitable areas calculated from the best model are in electronic supplementary material, table S8, and suitable areas within the IUCN protected areas for all types of models are in electronic supplementary material, figure S7.

**Figure 3.**
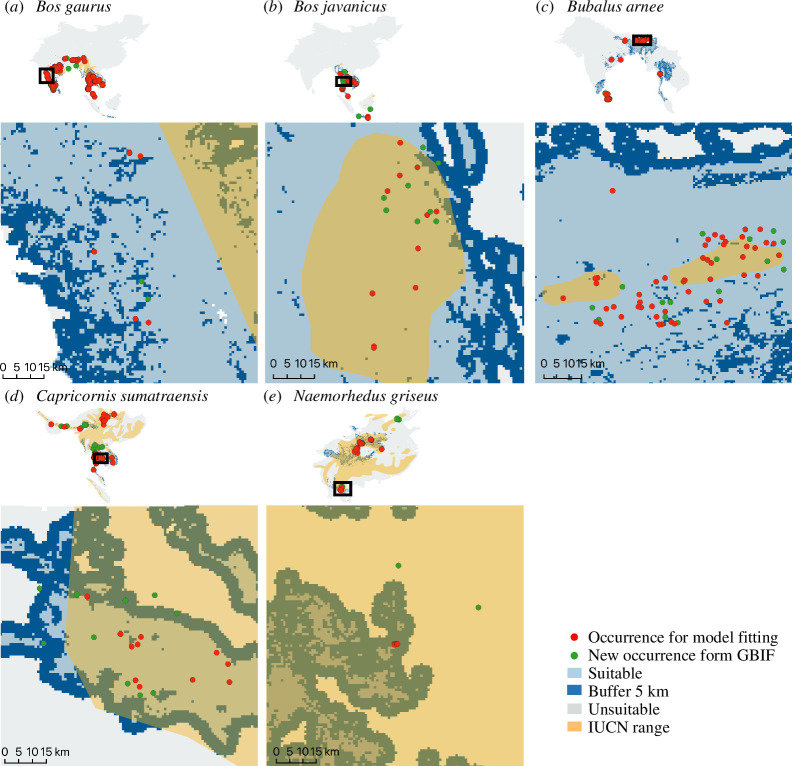
Model prediction testing for five bovid species (*a–e*) by calculating the percentage of the out-of-sample points that fall inside the predicted suitable areas (blue). The model fitting datasets (red) were mainly within the suitable areas compared with the new occurrence dataset (green). IUCN ranges show greater areas than the predictions for mainland serow and Chinese goral. Some occurrence data points were distributed outside both the model-predicted suitable area and the IUCN range.

Our model’s out-of-sample predictions with new species occurrences demonstrated a higher prediction accuracy within Thailand than the entire distribution, and this was further improved by including 5 km buffer zones, with the exception of Chinese goral, which exhibited poor accuracy across all scales (table 3 and figure 3). Implementing a buffer zone improved the accuracy of all four remaining species. For the large herbivore species, gaur, banteng and wild water buffalo, the model cropped to Thailand showed a higher accuracy (greater than 80%) compared with the entire distribution (approx. 60%–80%). We selected only model predictions with an accuracy percentage greater than 80% for further analyses. Out-of-sample points that lay outside suitable areas showed a mean distance to the nearest suitable area of around 1–6 km (table 4), which is within the possible movement range of these species. As a result, three species, including gaur, banteng and wild water buffalo, were retained, while two species, mainland serow and Chinese goral, were excluded from the rest of the study. Furthermore, we cropped the entire distribution to focus only on the results within Thailand as the amount of data collected and model predictions were higher compared with the entire species’ distributions. The distributions for all species can be found in electronic supplementary material, figures S3 and S4.

**Table 3.** Comparison of the accuracy of the selected best models^a^ in predicting out-of-sample data for the entire accessible areas range and Thailand.

	total	no buffer			buffer			
		unsuitable	suitable	accuracy %	unsuitable	suitable	buffer 5 km	accuracy %
entire accessible areas								
*B. gaurus* (Gaur)	221	85	136	62	23	136	62	90
*B. javanicus* (Banteng)	12	4	8	67	2	8	2	83
*B. arnee* (Wild water buffalo)	35	4	31	89	0	31	4	100
*C. sumatraensis* (Mainland serow)	21	17	4	19	7	4	10	67
*N. griseus* (Chinese goral)	10	9	1	10	7	1	2	30
Thailand								
*B. gaurus* (Gaur)	52	8	44	85	2	44	6	96
*B. javanicus* (Banteng)	10	2	8	80	0	8	2	100
*B. arnee* (Wild water buffalo)	1	0	1	100	0	1	1	100
*C. sumatraensis* (Mainland serow)	14	9	5	36	2	4	8	86
*N. griseus* (Chinese goral)	2	2	0	0	2	0	0	0

^a^ The best model for gaur is No MSDM-LA, banteng MSDM-LA, wild water buffalo and Chinese goral MSDM-SSA and mainland serow No MSDM-SSA.

**Table 4.** Nearest distance from out-of-sample points to suitable area.

species	point	distance (km)		
		min	mean	max
Gaur	52	0.0047	1.54	22.4
Banteng	10	0.0323	4.72	39.9
Wild water buffalo	1	0.811	0.811	0.811
Mainland serow	14	0.00668	6.07	38.1
Chinese goral	2	0.147	1.54	2.93

### 3.2. Identifying priority areas for conservation

Most predicted suitable habitats in protected areas are located in IUCN category Ia (Strict nature reserve), Ib (Wilderness area) and II (National Park) areas for all species, while IUCN category V (Protected landscape or seascape) has the least. Overall, more than half of the species’ suitable habitat is not under any form of protection defined by the WDPA (electronic supplementary material, table S8 and figure S8). The proportion of the suitable area in each WDPA from the best models for both the SSA and LA model ensembles for each species are presented in electronic supplementary material, figures S9 and S10.

**Figure 4.**
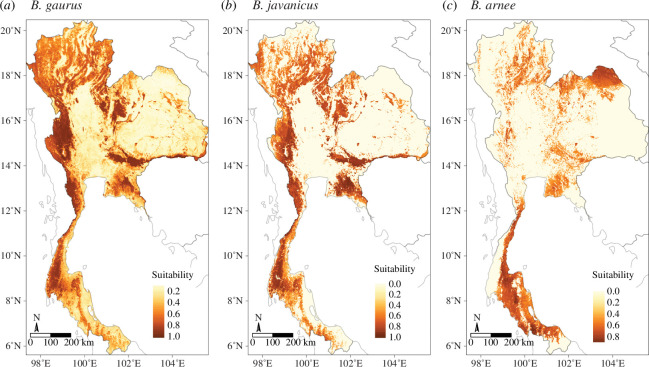
Habitat suitability prediction maps of three wild bovids species in Thailand: gaur (*B. gaurus*), banteng (*B. javanicus*) and wild water buffalo (*B. arnee*) species (*a–c*) using the best model from the weighted average ensemble. The value ranges from 0 to 1: yellow represents low suitability and dark brown represents high suitability. Interactive maps are provided in the electronic supplementary materials [65].

**Figure 5.**
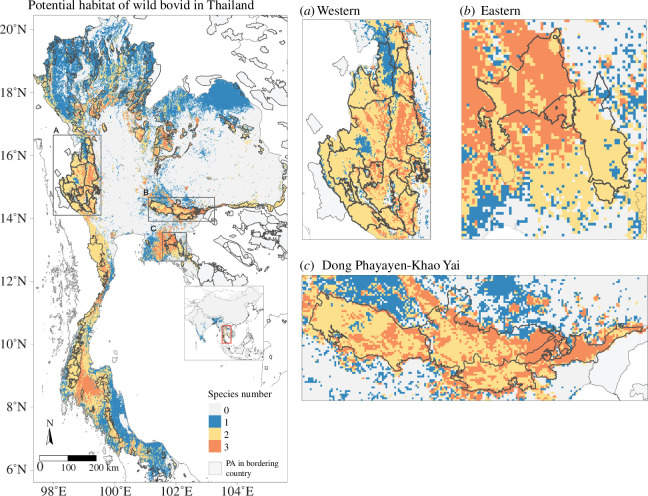
Estimated species richness of three wild bovids in Thailand. The species are gaur, banteng and wild water buffalo. Frames (*a–c*) focus on (*a*) Western Forest Complex (WEFCOM), (*b*) Dong Phayayen-Khao Yai Forest Complex (DPKY-FC) and (*c*) Eastern Forest Complex, where there are overlapping predicted suitable areas of all species (*n* = 3). Western, Dong Phayayen-Khao Yai and Eastern forests have suitable areas for gaur, banteng and wild water buffalo both inside PAs and in the surrounding areas.

In Thailand, we identified that more than 80% of the predicted suitable area larger than the species home range for gaur was located in 118 PAs covering 74 268 km^2^ (15% of Thailand), for banteng within 77 PAs covering 45 555 km^2^ (9% of Thailand), and for wild water buffalo within three PAs covering 559 km^2^ (0.1% of Thailand). A high proportion of the predicted suitable area for gaur and banteng is in Thungyai Naresuan, Kaengkrachan and Huai Kha Khaeng, and for wild water buffalo in Phu Wua WS and Dong Yai WS in eastern DPKY-FC (figures 4 and 5). The hotspots for all five species can be found in electronic supplementary material, figure S11.

We found that the highest percentage of predicted suitable areas comprised mixed deciduous forest for all species, followed by evergreen forest for gaur and banteng, and dry dipterocarp forest for wild water buffalo. We found a percentage of non-forest areas identified from the total suitable for three species: wild water buffalo (71%), banteng (33%) and gaur (24%). For more details of forest types of the suitable areas, see table 5 and electronic supplementary material, figure S12.

**Table 5.** The suitable areas of five bovid species classified by forest type in Thailand.

forest types	gaur		banteng		wild water buffalo		mainland serow		Chinese goral	
	(*Bos gaurus*)		(*Bos javanicus*)		(*Bubalus arnee*)		(*Capricornis sumatraensis*)		(*Naemorhedus griseus*)	
	km^2^	%	km^2^	%	km^2^	%	km^2^	%	km^2^	%
bamboo forest	390	0.22	348	0.19	178	0.13	250	0.37	2	0.02
beach forest	3	—	8	—	26	0.02	1	—	—	—
dry dipterocarp dorest	11 119	6.26	12 876	7.13	7365	5.43	1415	2.07	3546	23.91
dry evergreen forest	20 730	11.68	19 209	10.63	5944	4.38	13 893	20.3	1027	6.93
freshwater swamp forest	66	0.04	134	0.07	24	0.02	—	—	—	—
mangrove forest	609	0.34	1072	0.59	1028	0.76	121	0.18	—	—
mixed deciduous forest	66 132	37.25	59 211	32.77	18 837	13.88	25 243	36.88	7347	49.54
moist evergreen forest	14 802	8.34	15 729	8.7	1975	1.46	12 213	17.84	—	—
montane forest	16 693	9.4	8497	4.7	812	0.6	7532	11	1878	12.66
peat swamp forest	49	0.03	2	—	201	0.15	—	—	—	—
pine forest	634	0.36	185	0.1	87	0.06	78	0.11	15	0.1
savannah	548	0.31	348	0.19	108	0.08	312	0.46	7	0.05
secondary forest	2017	1.14	1856	1.03	1189	0.88	602	0.88	153	1.03
teak plantation	846	0.48	1045	0.58	919	0.68	60	0.09	12	0.08
vegetation on pen rock platform	201	0.11	208	0.11	118	0.09	90	0.13	2	0.01
other plantations	37	0.02	42	0.02	29	0.02	9	0.01	—	—
non-forest area	42 649	24.02	59 923	33.16	96 883	71.38	6631	9.69	842	5.68
total	177 526	100	180 693	100	1 35 725	100	68 452	100	14 831	100

## 4. Discussion

We modelled the potential distribution for five threatened wild bovid species present in Thailand, distributed in East, South and Southeast Asia. Our aim was to build predictive models to identify conservation areas and potential species richness maps in their entire geographical distributions. However, the model predictions were more accurate for Thailand, where most of the data were collected for all species except Chinese goral (table 3), therefore, we focused our analysis on Thailand. Our models were able to predict the presence of out-of-sample observations well for three species, gaur, banteng and wild water buffalo throughout their entire distributions (greater than or equal to 62%), but not mainland serow or Chinese goral (less than or equal to 19%). We identified that suitable areas were fragmented and often (all greater than 50%) located outside PAs. Those suitable areas outside PAs could possibly be managed as corridors or buffer zones to connect currently fragmented bovid populations inside PAs, thereby enhancing long-term wild bovid conservation success [66,67]. When considering the minimum likely areas of continuous, connected suitable habitat needed to maintain populations in PAs in Thailand, some habitats might be suitable but excluded here because we only considered suitable patches greater in area than home ranges, yet smaller patches might be connected enough if animals can move between them. Future analyses should consider the necessary required areas and the movement of animals between patches or habitats and their connectivity. We recommend incorporating fragmentation analyses at finer scale resolutions into specific location analyses in future studies to understand habitat fragmentation and prioritize vulnerable areas to support effective habitat management [68].

Our study found that the most suitable areas for gaur were aligned with IUCN range assessments [19] and other studies that have confirmed species presences in Thailand [26], Myanmar [69], the Western Ghats in southwestern India and Manas WS in the Himalayan foothills [70]. Our study predicted larger gaur suitable habitats in Thailand inside (approx. 82 400 km^2^) and outside (95 000 km^2^) PAs than Prayoon *et al*. [26], who predicted 39 508 km^2^ of total suitable habitat. Choudhury [70] predicted larger gaur distributions in the Western Ghats, Central and North-eastern India than our predictions. These differences might be due to the changes in the percentage of the forest cover and the habitat destruction over the past two decades, which has reduced the suitable areas in some locations. Our predictions also used NDVI and land coverage fractions (electronic supplementary material, table S3) for predicting greenness, which may be useful for predicting vegetation quality and availability for ungulates [71]. However, NDVI makes it difficult to differentiate variation among vegetation types [72,73], such as between specific agricultural areas, grasslands and dense forest canopies. This may include vegetation types other than the species’ preferred habitats in suitable areas and predicted larger suitable areas in non-forest areas and non-PAs in our study, compared with Prayoon *et al*.’s study [26]. Other studies suggest that gaur does use crop plantations or human-made grasslands, which may increase the suitable areas in our prediction, even if these are not their natural habitats and lead to conflict between humans and gaur [74].

Our best model predicted larger suitable areas (446 075 km^2^) for banteng than the IUCN-SSC report released in 2010 (approx. 209 000 km^2^) [75]. We found a high percentage of predicted suitable areas in Eastern Plains Landscape (ELP) and Chhaeb WS in Cambodia; the former supports the likely largest banteng population globally [76]. However, our results showed low habitat suitability in Sundaic Southeast Asia, with just 2% of the total suitable area in Indonesia (mainly in Alas Purwo NP, Java) and 2% of the total suitable area in Malaysia. Banteng populations and habitats in Southeast Asian islands (Borneo, Java and Bali) are threatened due to hunting for horn and meat consumption and habitat loss [77]. In Thailand, we found high suitability similar to previous studies in eastern [78] and western forest complexes [79], including reintroduction areas in Salak Pra WS [21] and where recent recolonization by natural population movement has occurred in Mae Wong NP [80].

Wild water buffalo has been domesticated and bred as livestock, making it hard to distinguish between the free-grazing domestic buffalo and wild water buffalo, as domesticated animals may replace wild animals in suitable habitats and cause high suitable area prediction outside PAs, especially in overlapping habitats [81]. We estimate the highest percentages of suitable areas in Kaziranga NP in India, which currently has the largest population of wild water buffalo [14]. Grasslands and floodplain areas of Manas NP (500 km^2^) and Kaziranga NP (greater than 850 km^2^) in India contain the most suitable habitat and are the main population strongholds for wild water buffalo [62]. In Thailand, this type of habitat can be found in many places, but it is not often represented in protected areas. Wild water buffalo are only found in Huai Kha Kheang WS parts of the Western Forest Complex. Our model predicts that only 43% of Huai Kha Kheang Wildlife Sanctuary is suitable for this species, primarily because the floodplains are mainly situated close to the main river in the middle of the PA. Additionally, the population has remained constant for decades, which could be attributed to a single population group or constraints within suitable habitats.

The three selected species showed overlapping suitable areas in the Western Forest Complex, Eastern Forest Complex, and Dong Phayayen-Khao Yai Forest Complexes (DPKY-FC). These forest complexes encompass extensive areas of high wildlife biodiversity and diverse forest types, including several contiguous PAs situated at the borders of Cambodia and Myanmar. The Western Forest Complex is the largest conservation area in Thailand where these wild bovids still exist. The DPKY-FC maintains a high population of gaur as it is mainly covered by evergreen forests. The Eastern Forest Complex sustains a large population of banteng because most of the vegetation consists of deciduous and dipterocarp forest. Gaur uses a diversity of types of habitats and prefers denser canopy at higher elevations than banteng*,* which tends to inhabit dry and open habitats such as dry dipterocarp and deciduous forests [82,83]. Wild water buffalo also shares overlapping areas with these two species, despite its distribution being found exclusively in Huai Kha Khaeng Wildlife Sanctuary. We recommend protecting these important suitable habitats to ensure the protection of wild bovids. This may involve implementing active patrolling to reduce illegal intrusions, snare removal and habitat management based on their diet diversity [84]. Additionally, one option to maintain wild water buffalo populations is to reintroduce them into their historical range, from which they have been extirpated. This method could be evaluated by combining predicted suitable areas with several important factors such as vegetation types, forage biomass, carrying capacity and hunting pressure [85].

In this study, we included all subspecies data points in our model ensembles as we aimed to extrapolate and predict the entire range of species’ habitat suitability, but this may increase uncertainty [86]. The five bovids have multiple subspecies, including three subspecies of gaur [19], banteng [87], wild water buffalo [14] and mainland serow [88], and two subspecies of Chinese goral [89]. Subspecies may vary in niche, climate and biological interactions that could affect the model predictions. The low habitat suitability we found in Borneo for banteng could be because climatic and geographic conditions differ for *Bos javanicus lowi* compared with those in mainland Asia, affecting model transferability across different regions [90]. Equally, Mori *et al*. [88] suggest that Chinese goral (*N. griseus*) should be reclassified within Brown goral (*N. goral*) together and Burmese goral (*N. evansi*) that together with *N. griseus* should be split to become a unique species. Future analyses must consider these taxonomic reclassifications. However, we modelled species-level habitat suitability, rather than the subspecies, as we assume that there is less likely to be habitat and environmental variation at the subspecies level for these bovids [91].

We found that using the MSDM OBR technique showed a better predicted suitable area of the ecological niche, closer to the real distribution for species with more restricted ranges like banteng, wild water buffalo and Chinese goral, with higher performance TSS values compared with No MSDM models. We recommend restricting the accessible area for predicting wild water buffalo potential habitat to reduce overprediction caused by overlapping areas with domestic water buffalo.

We also used ensemble approaches, to obtain better predictive performances than from any single model type, but further analyses could also look at individual model results using different parameters, such as differing pseudo-absence background point ratios. The equal ratio of presence to pseudo-absence (1 : 1 ratio) has been used in several types of models like general linear models, artificial neural networks and Maxent models, and it is also recommended for use in ensemble models when dealing with small sample sizes [92].

We acknowledge sampling deficiencies across the regions. We had fewer occurrences in Vietnam, Laos, Myanmar and Indonesia compared with Thailand, from which a large number of our data points came (30 512 points in Thailand, 3152 points outside Thailand, table 2). Occurrence data based on data accessibility may have sampling bias, particularly with clustered points for gaur, banteng and mainland serow. We minimized these biases through spatial thinning [93]. Since we found large amounts of suitable areas outside of Thailand, we suggest that future studies should focus on monitoring bovid populations in other countries, especially in India and Myanmar. However, because of this and the model performance, we focused on Thailand. For both banteng and wild water buffalo, we also observed that there was higher suitability predicted by our models for areas of montane forest type (5% for banteng) and high elevation (over 1300 m) and slope (9°) than we consider likely to be highly suitable for these species. In our raw data, the highest elevation for banteng is 800 m and for wild water buffalo 684 m. We examined the data and observed that the most important predictors are bioclimatic variables, rather than topographic variables (elevation, slope etc.), which showed a lower percentage of contribution to the model building (see electronic supplementary material, table S6 and figures S2 (panel D) and S11).

We are aware of the limitations of using the WDPA dataset, as a previous study has found a lower proportion in WDPA-protected areas compared with China’s National Nature Reserves (CNNR) [94]. However, we used the WDPA dataset as it provides data for the entire distribution at a resolution that is appropriate for our analysis and for the consistency of modelling and interpreting the results. Moreover, for Thailand, our main area of study, the WDPA database’s forest areas and categories are mostly consistent with Thai protected areas (e.g. national parks, wildlife sanctuaries and non-hunting areas) and this database has been used for conducting species distribution modelling [95] and evaluating the effectiveness of protected areas in Thailand [96].

Missing data has probably impacted some results. The model TSS values for endangered banteng and Chinese goral are over 0.8, yet our models predict no suitable areas in parts of Indonesia (east and central Kalimantan [77]) for banteng, and in China (e.g. Beijing and northeast Inner Mongolia [97]) for Chinese goral from which these species have been reported. This would probably be improved if more spatial data were available for these species. Recent surveys of gaur and banteng in China suggest gaur are present, but banteng is extinct [98]. Our models have not predicted highly suitable areas for gaur in China, though there are suitable areas in the north of Myanmar close to South Yunnan, whereas for banteng our findings are similar, with very low predicted suitable areas for banteng in China (5–20 km²; electronic supplementary material, table S8)—a notably small area compared with the entire country. However, we predicted the largest suitable areas for Chinese goral (approx. 285 000 km^2^) and mainland serow (approx. 60 000 km^2^) in China, which are within the range of those reported [99]. We used a new dataset of species occurrences to assess our model’s performance with a 5 km buffer zone, aiming to enhance modelling accuracy. Given these species have quite large home ranges and daily movements, adding a buffer to represent this movement unsurprisingly leads to better model predictions for all species, but most notably for mainland serow, changing the out-of-sample prediction from 19% to 67% for the entire region and 36%–86% for Thailand. The buffer zone may indicate the utilization of unsuitable areas of the species near forested regions, such as secondary forests, agricultural areas or water resources, which possibly extend these buffer areas from the protected areas to enhance the wildlife protection.

The spatial restriction method, OBR, can be sensitive to the distribution of occurrence data because it keeps predicted suitable areas close to the occurrence locations. This may lead to the exclusion of potentially suitable areas driven by a lack of occurrence data in those areas. For example, the wild water buffalo No MSDM predicted potentially suitable habitat around the Sre Pok Wildlife Sanctuary in Cambodia where the species is distributed [76], but after the spatial restriction (MSDM), this potential habitat was excluded as we lack occurrence data in Cambodia. Although our study showed slightly different TSS values between two different accessible area extents, we encourage testing the different accessible areas as it affects the model results [6]. Moreover, model performance varied with accessible area sizes and spatial restrictions, emphasizing the need for careful accessible area definition in ecological modelling [5]. Further, future analyses may try to better account for the current presence of species by accounting for factors such as hunting using other proxies, such as other human-disturbance metrics like distance from roads [100].

## 5. Conclusion

Our study provided an overview of the suitable remaining habitat for threatened bovid species at a regional scale using high-resolution environmental variables and species occurrence data from multiple observation methods. Our predictions showed that the suitable areas are small and fragmented for all species, and more than 50% of suitable areas are outside of protected areas. Those suitable areas outside PAs could possibly become efficient conservation areas, such as forest corridors or buffer zones to connect fragmented bovid populations and enhance long-term habitat conservation. Our predictions may inform conservation actions to avoid further defaunation of wild bovidae such as the management of human–wildlife conflicts and habitat quality for long-term species survival.

## Data Availability

Data and relevant code for this research work are stored in GitHub [65]. Bovidae and have been archived within the Zenodo repository [101]. Supplementary material is available online [102].
